# Intracranial Pressure-Guided Therapy in 3,4-Methylenedioxymethamphetamine (MDMA)-Induced Cerebral Edema: A Case Report

**DOI:** 10.7759/cureus.90328

**Published:** 2025-08-17

**Authors:** Haytham Khalifa, Mohamed Abdulmajeed

**Affiliations:** 1 Intensive Care Unit, National Hospital for Neurology and Neurosurgery, London, GBR; 2 General Internal Medicine, Luton and Dunstable University Hospital, Luton, GBR

**Keywords:** cerebral edema, ecstasy toxicity, hyponatremia, icp monitoring, mdma, neurocritical care, recreational drug, serotonin syndrome, stimulant overdose, water intoxication

## Abstract

3,4-Methylenedioxymethamphetamine (MDMA), commonly called ecstasy, is a synthetic stimulant popular among young adults. Although its psychoactive effects are sought after, MDMA can lead to serious complications, including neurological and cardiovascular crises. Severe toxicity, although uncommon, may result in cerebral edema, hyponatremia, seizures, or death. We describe the case of a 21-year-old woman who collapsed after taking MDMA and drinking large volumes of water. On arrival at the hospital, she was found to have significant cerebral edema and low sodium. After endotracheal intubation and transfer to a tertiary center, she was managed with intracranial pressure (ICP) monitoring and careful correction of hyponatremia. She made a full recovery and was discharged from intensive care within four days. This report highlights how ICP-guided management can help optimize care and reduce risks in patients with MDMA-related cerebral edema.

## Introduction

3,4-Methylenedioxymethamphetamine (MDMA), commonly called ecstasy, is a synthetic amphetamine derivative widely used as a recreational drug among young people [1]. It produces feelings of heightened energy, emotional warmth, and social connectedness [2]. On a molecular level, MDMA increases the release and inhibits the reuptake of serotonin, dopamine, and norepinephrine [3]. These effects, along with its ability to stimulate antidiuretic hormone release, contribute to water retention and impaired water excretion [4]. When combined with excessive fluid intake, this can lead to severe hyponatremia and brain swelling [4].

After ingestion, MDMA is absorbed through the gut, usually producing effects within an hour and peaking at two hours. The liver metabolizes the drug, and its active metabolites can prolong its clinical impact beyond the detectable drug levels [5,6]. Although most users recover uneventfully, a subset develop life-threatening complications requiring critical care.

In severe cases of drug-induced cerebral edema, intracranial pressure (ICP) monitoring may offer continuous, real-time guidance for therapy, yet its application in MDMA-related hyponatremia is rarely reported, making this case an opportunity to explore its potential benefits.

## Case presentation

A healthy 21-year-old woman was brought to the emergency department after collapsing at a nightclub. According to her partner, she had ingested MDMA about two hours earlier and consumed several liters of water. At the scene, paramedics noted that she was confused and agitated, with a Glasgow Coma Scale (GCS) score of 9/15. Her pulse was 118 beats per minute, blood pressure 130/80 mmHg, respiratory rate 24 breaths per minute, oxygen saturation 98%, and temperature 37.2°C.

While being assessed in the emergency department, her mental state deteriorated further, and her GCS dropped to 7/15. She was sedated, intubated, and mechanically ventilated. Blood tests revealed a serum sodium of 123 mmol/L and mild respiratory alkalosis on arterial blood gas (Table 1). Creatinine kinase was slightly elevated, and urine toxicology was positive for amphetamines.

**Table 1:** Serial laboratory results (ICU days 0-3)

Parameter	Reference range	Day 0	Day 1	Day 2	Day 3
White blood cell (WBC) (×10⁹/L)	4.0-11.0	9.43	8.01	10.26	11.30
Hemoglobin (g/dL)	120-160	133	108	123	112
Platelets (×10⁹/L)	150-400	246	264	215	232
Sodium (Na⁺) (mmol/L)	135-145	123	127	138	142
Potassium (K⁺) (mmol/L)	3.5-5.1	4	5,9	4.1	4.4
Urea (mmol/L)	2.5-7.8	3.2	3.6	3.8	2.8
Creatinine (µmol/L)	45-90	57	53	70	60
Alanine aminotransferase (ALT) (U/L)	7-56	27	19	18	24
Creatine kinase (U/L)	26-192	721	619	456	218
C-reactive protein (CRP) (mg/L)	<5	0.6	4.2	49	74
Arterial pH	7.35-7.45	7.31	7.39	7.39	7.47
pCO₂ (kPa)	4.7-6.0	6.8	4.38	4.88	4.56
pO₂ (kPa)	10.0-13.3	73.3	20.2	10.2	11.6
HCO₃⁻ (mmol/L)	22-29	22.3	21	22.6	26.1
Lactate (mmol/L)	0.5-2.2	0.8	0.6	0.4	0.4

**Figure 1:**
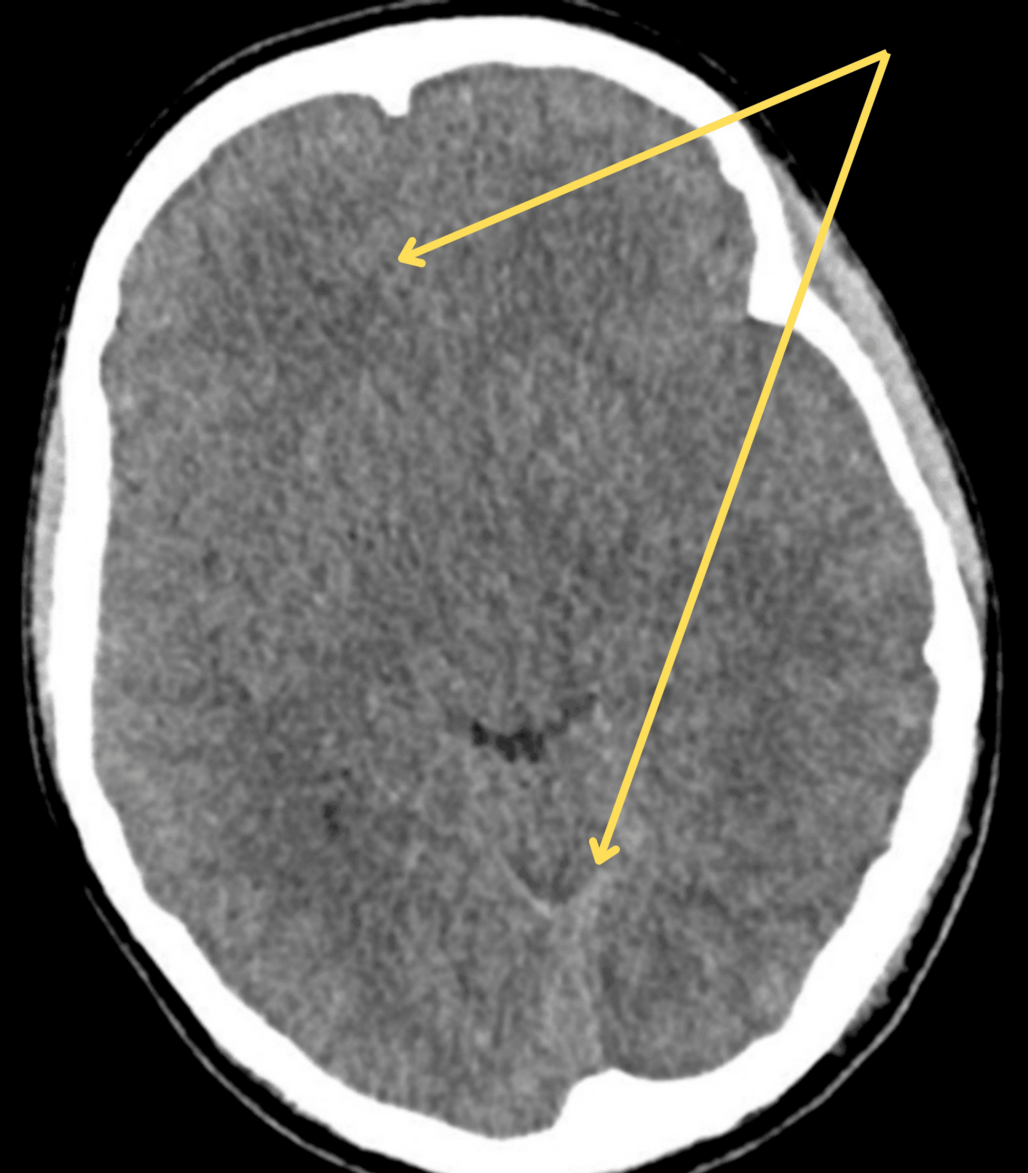
Non-contrast computed tomography (CT) of the brain showing diffuse cerebral edema with effacement of sulci and compressed ventricles, consistent with early signs of raised intracranial pressure There are marked effacement of cortical sulci, compressed lateral ventricles, and reduced gray‑white matter differentiation, findings consistent with elevated intracranial pressure.

A computed tomography (CT) scan of the head showed diffuse brain swelling without hemorrhage or midline shift (Figure 1). She was started on hypertonic saline and fluid restriction. Despite initial management, her poor neurological response raised concerns about increased ICP. She was transferred to a tertiary care ICU.

**Figure 2:**
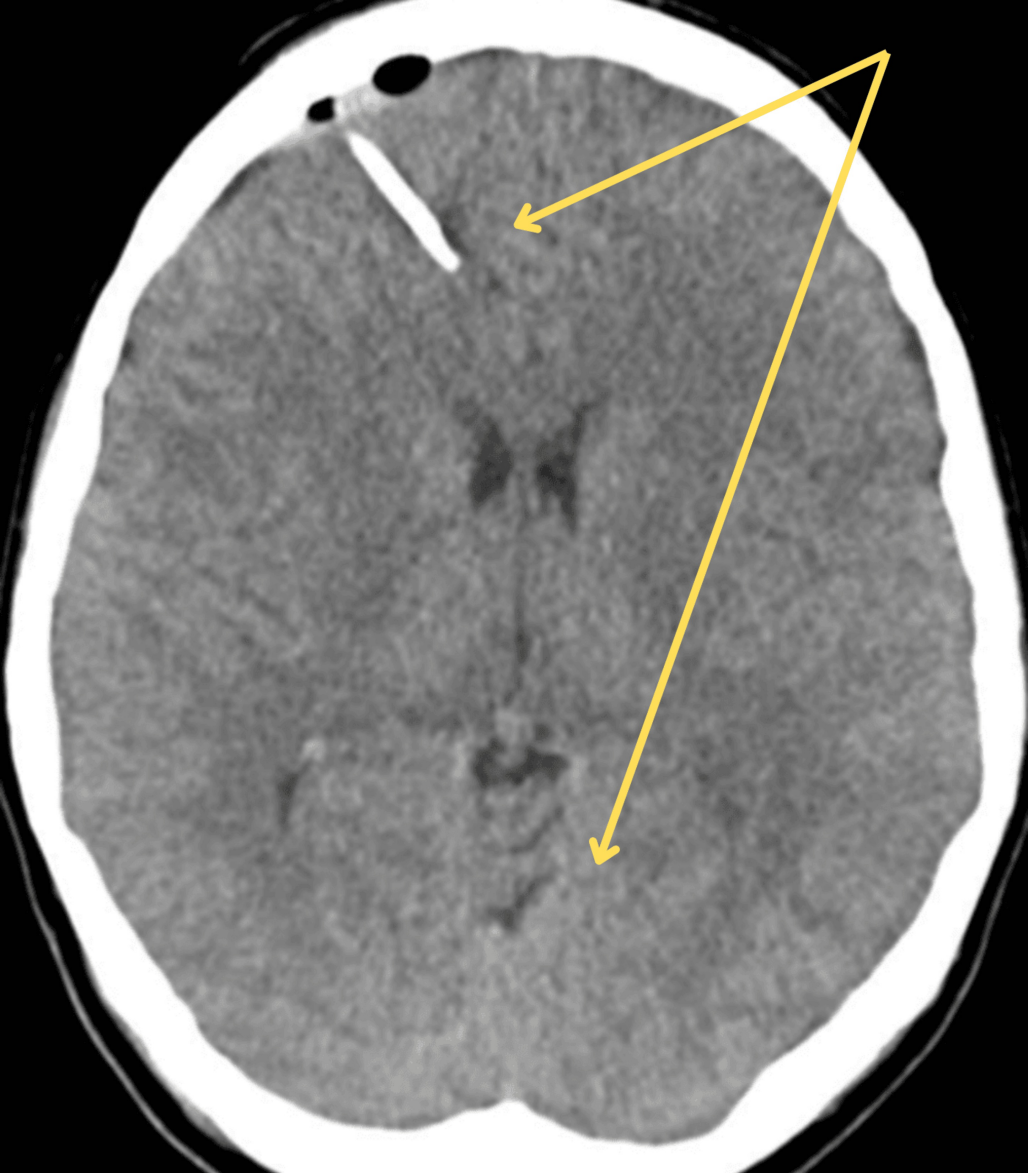
Follow‑up non‑contrast axial computed tomography (CT) of the brain after 24 hours of therapy showing reduction of cerebral edema with the restoration of ventricular and sulcal anatomy compared to the admission scan

At the tertiary center, a frontal ICP monitoring bolt was inserted, revealing pressures between 8 and 10 mmHg, with transient elevations during procedures. Sedation was adjusted to maintain cerebral protection without unnecessary deep sedation, guided by Patient State Index monitoring. Hypertonic saline was slowly reduced over 48 hours, ensuring sodium levels rose at a safe rate. Repeat imaging on the second day showed significant improvement in cerebral edema (Figure 2).

By the third ICU day, sedation was discontinued, and she regained full consciousness. She was extubated on the fourth day, with the ICP monitor removed. After two more days of observation on the general ward, she was discharged home in good health.

## Discussion

MDMA toxicity can result in life-threatening complications, including cerebral edema secondary to hyponatremia. While this pathophysiology is well described [1-4], the use of ICP monitoring as a therapeutic guide in such cases is not routinely implemented and lacks standardized guidance. Our case highlights the potential utility of ICP-guided care in optimizing treatment and improving outcomes in MDMA-induced cerebral edema.

MDMA exerts its effects by increasing serotonin, dopamine, and norepinephrine levels while also stimulating antidiuretic hormone secretion [2,3]. When taken in high doses or alongside excessive water consumption, as in this case, patients are at risk of developing acute water intoxication and severe hyponatremia, which can lead to cerebral edema and raised ICP [3,4]. Rapid shifts in plasma osmolality cause water to enter neurons and glial cells, leading to brain swelling and potential herniation [5].

Treatment of MDMA-induced cerebral edema typically focuses on fluid restriction and hypertonic saline [5]. However, overcorrection of sodium may cause osmotic demyelination syndrome (ODS), which carries its own morbidity. Guidelines recommend increasing serum sodium by no more than 8-10 mmol/L in the first 24 hours [5]. In our case, real-time ICP monitoring enabled the dynamic tailoring of hypertonic therapy while avoiding over-sedation and allowing prompt neurological assessment.

While ICP monitoring is well established in the setting of traumatic brain injury (TBI), its application in toxic-metabolic encephalopathy, such as MDMA toxicity, is still evolving. In our case, the rationale for ICP monitoring was based on the patient's high-risk trajectory rather than the initial ICP reading. A low opening pressure does not exclude the possibility of later surges during osmotic shifts or care-related stimulation. Furthermore, the need for ongoing sedation precluded reliable neurological examinations, and neuroimaging alone could not provide continuous, real-time feedback. Non-invasive modalities, such as transcranial Doppler or optic nerve sheath diameter measurement, were considered but deemed insufficient to guide the fine-tuning of sodium correction, sedation weaning, and hyperosmolar therapy in this rapidly evolving scenario.

Recent literature has begun to support the use of ICP monitoring in such contexts to maintain pressures below 20-25 mmHg, correlating with improved outcomes [6]. In our patient, ICP values remained between 8 and 10 mmHg with transient elevations, and this feedback guided sedation and therapy adjustments precisely.

Without invasive monitoring, clinicians often rely on surrogate markers, like CT imaging or neurological exams, to assess cerebral edema. These methods are delayed or limited when the patient is sedated and intubated. In contrast, direct ICP measurement provided continuous physiologic feedback, allowing the safe weaning of sedation and earlier extubation. The patient recovered neurologically and was discharged from the ICU within four days.

It is also worth emphasizing that young women are particularly susceptible to MDMA-related hyponatremia. Estrogen has been shown to reduce Na⁺/K⁺ ATPase activity in astrocytes, impairing the brain's ability to regulate cell volume in hyponatremic states [3,4]. Lower body mass and a stronger antidiuretic hormone response may further contribute to vulnerability in this population. Public health strategies should address this demographic through education about the dangers of MDMA, excessive fluid intake, and the importance of early medical attention when symptoms like confusion or seizures occur.

This case contributes to the growing body of evidence supporting the use of ICP monitoring beyond trauma, demonstrating that it can guide therapy, reduce iatrogenic risks, and shorten ICU stay in selected cases of drug-induced cerebral edema.

## Conclusions

ICP monitoring may be a valuable adjunct in selected cases of MDMA-induced cerebral edema, particularly when neurological assessment is limited and there is concern for dynamic intracranial changes. In this case, continuous ICP monitoring helped guide individualized therapy, informed the pace of sodium correction, and supported timely sedation weaning. However, these findings should be interpreted with caution, given the single-patient nature of the report and the relatively normal ICP readings observed. Further research and accumulation of similar cases are needed before broad recommendations can be made regarding the routine use of ICP monitoring in toxic-metabolic encephalopathy.
